# Kidney injury molecule-1 is a potential receptor for SARS-CoV-2

**DOI:** 10.1093/jmcb/mjab003

**Published:** 2021-01-25

**Authors:** Chen Yang, Yu Zhang, Xia Zeng, Huijing Chen, Yuchen Chen, Dong Yang, Ziwei Shen, Xiaomu Wang, Xinran Liu, Mingrui Xiong, Hong Chen, Kun Huang

**Affiliations:** 1 School of Pharmacy, Tongji Medical College, Huazhong University of Science & Technology, Wuhan 430030, China; 2 Tongji-RongCheng Biomedical Center, Tongji Medical College, Huazhong University of Science & Technology, Wuhan 430030, China

**Keywords:** SARS-CoV-2, COVID-19, kidney diseases, kidney injury molecule-1, coronavirus

## Abstract

COVID-19 patients present high incidence of kidney abnormalities, which are associated with poor prognosis and mortality. The identification of SARS-CoV-2 in the kidney of COVID-19 patients suggests renal tropism of SARS-CoV-2. However, whether there is a specific target of SARS-CoV-2 in the kidney remains unclear. Herein, by using *in silico* simulation, co-immunoprecipitation, fluorescence resonance energy transfer, fluorescein isothiocyanate labelling, and rational design of antagonist peptides, we demonstrate that kidney injury molecule-1 (KIM1), a molecule dramatically upregulated upon kidney injury, binds with the receptor-binding domain of SARS-CoV-2 and facilitates its attachment to cell membrane, with the immunoglobulin variable Ig-like (Ig V) domain of KIM1 playing a key role in this recognition. The interaction between SARS-CoV-2 receptor-binding domain and KIM1 is potently blockaded by a rationally designed KIM1-derived polypeptide AP2. In addition, our results also suggest interactions between KIM1 Ig V domain and the receptor-binding domains of SARS-CoV and MERS-CoV, pathogens of two severe infectious respiratory diseases. Together, these findings suggest KIM1 as a novel receptor for SARS-CoV-2 and other coronaviruses. We propose that KIM1 may thus mediate and exacerbate the renal infection of SARS-CoV-2 in a ‘vicious cycle’, and KIM1 could be further explored as a therapeutic target.

## Introduction

The World Health Organization has announced the coronavirus disease 2019 (COVID-19) as a pandemic (Guan et al., 2020). Severe acute respiratory syndrome coronavirus 2 (SARS-CoV-2), the pathogen of COVID-19, belongs to the beta-coronavirus genus that also includes SARS-CoV and Middle East respiratory syndrome coronavirus (MERS-CoV) (Shahrajabian et al., 2020). SARS-CoV-2, SARS-CoV, and MERS-CoV mainly target respiratory systems to primarily manifest with respiratory illness (Shahrajabian et al., 2020). Notably, reports about renal involvement among patients infected, as well as identification of viral infection in the kidney suggested that these coronaviruses may directly infect the kidney (Ding et al., 2004; Eckerle et al., 2013; Braun et al., 2020; Su et al., 2020).

Kidney impairment in hospitalized COVID-19 patients is common, and we and others have reported its association with severe inflammation, poor clinical progress, and high in-hospital mortality (Pei et al., 2020; Hirsch et al., 2020; Chen et al., 2020; Yang et al., 2020). High incidence of acute kidney injury (AKI) (56.9%) among patients with COVID-19 has been observed (Fisher et al., 2020). Importantly, the presence of infective SARS-CoV-2 has been confirmed in the kidney, especially in renal epithelial cells; and a post-mortem study suggested the renal tropism of SARS-CoV-2, which was detected in the kidneys of 72% of COVID-19 patients with AKI (Braun et al., 2020). Among multi-organ manifestations in COVID-19 patients, apart from the lung, the kidney is highly vulnerable to the virus, and renal dysfunctions are closely associated with high mortality, with the underlying molecular mechanisms remaining unclear.

SARS-CoV-2 invasion initiates from binding with cellular membrane receptors via its spike protein (Shahrajabian et al., 2020). Presently, angiotensin-converting enzyme 2 (ACE2), which is enriched in the kidney and also the target for SARS-CoV, is the only well-recognized receptor for SARS-CoV-2 (Shang et al., 2020). Responsible for receptor recognition, SARS-CoV-2 spike protein (SARS-CoV-2-S) consists of subunits S1 and S2 (Figure 1A), and the receptor-binding domain (RBD) in S1 binds ACE2 to initiate the fusion of S2 with cell membrane and subsequent cell entry (Shang et al., 2020). Although comparison on the level of ACE2 between SARS-CoV-2 infected and normal kidneys has not been reported, decreased level of ACE2 upon coronavirus infection in lung has been observed (Kuba et al., 2005), therefore, the renal tropism of SARS-CoV-2 and associated kidney injury seem unlikely associate with the level of ACE2. In addition, we recently reported that the administration of ACE2 inhibitors showed no association with clinical outcomes among COVID-19 patients (Chen et al., 2020). Given that viral cell entry may involve multiple transmembrane receptors (Lan et al., 2020), we speculate that additional receptors may mediate the renal infection of SARS-CoV-2.

Kidney injury molecule-1 (KIM1) is primary expressed in kidney and drastically up-regulated in injured kidney proximal tubule upon injury, and plays crucial roles in inflammation infiltration and immune responses (Rong et al., 2011). Structurally, KIM1 consists of immunoglobulin variable Ig-like (Ig V) domain, mucin domain, transmembrane domain and cytosolic domain. Among them, Ig V domain is required for virus binding and internalization, such as the entry of Ebola and Dengue viruses (Yuan et al., 2015; Dejarnac et al., 2018). Here, we investigated whether KIM1 is a binding target of SARS-CoV-2 that mediates its kidney invasion.

## Results

### *Expression profiles of KIM1 and ACE2 in human tissues*

To elucidate KIM1 and ACE2 enrichment in tissues, the transcriptome and histology-based protein expression data from the Tissue Atlas of Human Protein Atlas were collected. The top 10 tissues with mRNA and protein abundance of KIM1 and ACE are listed in Supplementary Figure S1A–F. Notably, KIM1 and ACE2 co-expressed in the kidney, colon, rectum, testis, and gallbladder (Supplementary Figure S1C and F), which are all among the target organs of SARS-CoV-2 (Cha et al., 2020), implicating a close correlation of KIM1 with COVID-19 manifestations.

*Molecular dockings reveal the interaction between SARS-CoV-2-RBD and KIM1 Ig V*

The primary sequences of SARS-CoV-RBD and SARS-CoV-2-RBD share high similarity (62.93%), with 9 of 14 ACE2-contacting residues conserved in both RBDs (Figure 1B). In comparison, MERS-CoV-RBD, which recognizes DPP4 (Li et al., 2020c), shows low similarity with SARS-CoV-RBD and SARS-CoV-2-RBD (17.07% and 14.86%, respectively; Figure 1B).

*Kim1* is drastically upregulated in the kidneys of ischemia–reperfusion (I/R)- or cisplatin-injured mice, while only mild changes of *Ace2* were observed (Figure 1C). Among four domains of KIM1 (Figure 1D and E), Ig V domain is responsible for virus binding (Yuan et al., 2015; Dejarnac et al., 2018), and molecular dynamic docking was thus conducted to investigate its binding with SARS-CoV-2-RBD.

Docking and structural information of SARS-CoV-2-RBD and KIM1 Ig V complex are provided in Supplementary Figures S1G–I and S2. Phe338, Val367, Ser371, Phe374, and Trp436 of SARS-CoV-2-RBD contact Leu54, Phe55, Gln58, Trp112, and Phe113 of KIM1 Ig V (Figure 2A) and lead to a combined binding free energy of -35.64 kcal/mol (Table 1; Supplementary Table S1), which is lower than that of SARS-CoV-2-RBD and ACE2 (-50.60 kcal/mol) but comparable to that of SARS-CoV-RBD and ACE2 (-38.3 kcal/mol) (Li et al., 2020c). Since ACE2 is recognized as a key receptor for SARS-CoV-RBD (Li et al., 2020c), a strong interaction between SARS-CoV-2-RBD and KIM1 is suggested (Table 1; Supplementary Table S1). Notably, the different binding regions of SARS-CoV-2-RBD to KIM1 and to ACE2 indicated by our data suggest that KIM1 and ACE2 may synergistically mediate SARS-CoV-2 invasion (Figure 2B).

Clinically, mutations in SARS-CoV-2-S have been identified (Supplementary Figure S3 and Table S2), and COVID-19 cases carrying V367F mutation in SARS-CoV-2-S, which contacting KIM1, have been reported (http://giorgilab.dyndns.org/coronapp/, summarized in Supplementary Figure S3B and C) (Mercatelli et al., 2020). Molecular mechanics generalized born surface area (MM-GBSA) analysis suggests that V367F mutation leads to enhanced binding with KIM1 (Supplementary Table S1), which may associate with clinical findings that V367F leads to enhanced infectivity of SARS-CoV-2 (Starr et al., 2020; Li et al., 2020a); further investigations on these clinical mutations will be important.

*SARS-CoV-RBD and SARS-CoV-2-RBD target the same binding pocket in KIM1*

Microarray data showed increased *Kim1* expression in SARS patients-derived peripheral blood mononuclear cells compared to healthy controls (GSE1739, Supplementary Figure S4A; Reghunathan et al., 2005). Considering the facts that SARS-CoV-RBD and SARS-CoV-2-RBD both invade the kidney (Ding et al., 2004; Braun et al., 2020) and share high homology (Figure 1B), we evaluated the binding potential of SARS-CoV-RBD with KIM1 (Supplementary Figures S4 and S5). Sharing the same binding pocket within KIM1 (contacting surface shown in Figure 2C), SARS-CoV-RBD binds to KIM1 Ig V at a combined free energy of -21.59 kcal/mol (Supplementary Tables S1 and S3), suggesting a relatively lower affinity to KIM1 than that of SARS-CoV-2-RBD (-35.64 kcal/mol), whereas an even weak interaction was found between MERS-COV-RBD and Ig V (-10.12 kcal/mol, Supplementary Table S1). Therefore, SARS-CoV-2-RBD showed the highest binding affinity to KIM1; moreover, SARS-CoV-RBD and SARS-CoV-2-RBD share the same binding pocket on the Ig V domain (Figure 2C).

*Intracellular interaction of SARS-CoV-2-RBD and KIM1 Ig V*

To confirm the binding between SARS-CoV-2-RBD and KIM1, endogenous and exogenous co-immunoprecipitation (co-IP) assays were performed (Figure 3A–D; Supplementary Figure S6A). The exogenous viral proteins (Flag-tagged SARS-CoV-2-S or SARS-CoV-2-RBD) were immunoprecipitated with KIM1 from cell lysates of human kidney tubular epithelial cell line HK-2 and HEK293T cells that transfected with corresponding plasmids (Figure 3B; Supplementary Figure S6A), indicating direct binding of KIM1 and SARS-CoV-2-RBD. Similar results were also obtained by fluorescence resonance energy transfer (FRET)-based assay (Karpova et al., 2006) using KIM1-cyan fluorescent protein (CFP) and SARS-CoV-2-RBD-yellow fluorescent protein (YFP) (Figure 3F–J). Unconjugated CFP and YFP were co-transfected as the negative control (Figure 3E, F, and J), and the interaction between KIM1-CFP and its ligand TIM4-YFP was also included as a positive control (Figure 3E, G, and J). As detected by fluorescence spectrophotometry and confocal microscopy, co-transfection of KIM1-CFP and SARS-CoV-2-RBD-YFP in HEK293T cells resulted in a robust FRET signal (Figure 3E and H), indicating intracellular interaction between KIM1 and SARS-CoV-2-RBD.

Since KIM Ig V is crucial in mediating viral receptor binding (Yuan et al., 2015; Niu et al., 2018), plasmids overexpressing full-length KIM1, the Ig V domain of KIM1, or truncated KIM1 without Ig V domain (ΔIg V) were respectively co-transfected with SARS-CoV-2-RBD into a stable

KIM1 knockout HK-2 cell line or HEK293T cells (Figure 3C and D; Supplemental Figure S6B and C). Knocking out KIM1 or deletion of Ig V domain abolished the binding between KIM1 and SARS-CoV-2-RBD (Figure 3C and D). The interaction between KIM1 Ig V and SARS-CoV-2-RBD was also verified by FRET-based assays, and no obvious FRET signal was observed in cells co-transfected with KIM1 ΔIg V-CFP and SARS-CoV-2-RBD-YFP (Figure 3I and J). These results together suggest that Ig V domain is crucial in mediating the interaction between KIM1 and SARS-CoV-2.

*KIM1 mediates cell attachment of SARS-CoV-2-RBD*

We next used fluorescein isothiocyanate (FITC) labelling to track SARS-CoV-2-RBD in human cells. For each indicated group, at least 100 cells from 5 fields under high power objective lens were included in assessment. We observed less binding signal of FITC-SARS-CoV-2-RBD on the surface of human renal cells when KIM1 was knocked out, while more intense signal when KIM1 was overexpressed (Figure 4A and B). In KIM1 knockout HK-2 cells, restoring full-length KIM1 and overexpressing Ig V both rescued binding signals of SARS-CoV-2-RBD on cell surface (Figure 4A), demonstrating the importance of KIM1 Ig V in mediating viral attachment. Moreover, knockout of KIM1 attenuated the cytotoxicity induced by SARS-CoV-2-RBD (Supplementary Figure S6D). Together, these results further confirm the crucial role of Ig V domain in mediating SARS-CoV-2 attachment to renal cells.

*A KIM1-derived peptide blockades cell attachment of SARS-CoV-2-RBD*

To competitively bind with SARS-CoV-2-RBD and inhibit its interaction with KIM1, we rationally designed two antagonist peptides based on SARS-CoV-2-contacting motifs in KIM1 (motif 1: Leu54, Phe55, Gln58; motif 2: Trp112, Phe113; Figure 5A). Peptide 1 (AP1) mimics motif 1, while peptide 2 (AP2) covers both motifs, with three glycine used as a flexible linker (Figure 5A). The binding free energy, which indicates binding between peptides and SARS-CoV-2-RBD, was provided in Supplementary Table S1. Both peptides did not show distinct cytotoxicity, and AP2 reduced SARS-CoV-2-RBD attachment to cell surface and protected against its cytotoxicity (Figure 5B–D). Moreover, AP2 significantly inhibited the interaction between KIM1 and SARS-CoV-2-RBD, indicated by the abolished FRET signal between KIM1 and SARS-CoV-2-RBD upon AP2 treatment (Figure 5E and F). Enhanced SARS-CoV-2-RBD binding and prolonged half-life are undergoing by optimizing the sequences or modifications of AP2 with the approaches we recently described (Wang et al., 2020). Since KIM1 is protective against AKI (Yang et al., 2015), our strategy is unlikely to interfere with the beneficial effects of KIM1 *in vivo*.

**Discussion**

To fight against COVID-19 pandemic, a deep understanding of how SARS-CoV-2 invades human cells is warranted. Studies have indicated direct infection of SARS-CoV-2 in the kidney in addition to the lung (Braun et al., 2020; Farkash et al., 2020). However, ACE2 remains the only well-recognized receptor that may mediate this invasion. Furthermore, the renal tropism of SARS-CoV-2 and associated kidney injury seem unexplainable by the relatively decreased level of ACE2 upon viral invasion (Kuba et al., 2005). Here, our study suggests that KIM1, a drastically upregulated biomarker for kidney injury (Yang et al., 2015), mediates SARS-CoV-2 kidney invasion as a receptor.

We also found that SARS-CoV-2-RBD binds to KIM1 with a higher affinity than that of SARS-CoV-RBD and MERS-COV-RBD, which probably underlies the stronger contagion of SARS-CoV-2 (Rabaan et al., 2020); therefore, the renal infection and the roles of KIM1 in these severe respiratory diseases worth revisiting. Notably, our results suggest distinct binding sites of KIM1 and ACE2 on viral RBD, thus it is worth investigating whether and how KIM1 and ACE2 co-mediate SARS-CoV-2 invasion in these organs. In addition, since KIM1 is endocytosed via clathrin-dependent pathways (Zhao et al., 2016), it would also be interesting to further explore the KIM1-dependent process after viral attachment to cell membrane.

ACE2 is the most well-studied receptor for SARS-CoV-2, yet it is not an ideal therapeutic target for COVID-19, since it is widely expressed in multiple organs and plays crucial roles in regulating blood pressure and preventing heart/kidney injury (Imai et al., 2005; Li et al., 2020d). In contrast, KIM1 has stronger association with kidney function and is highly expressed only after renal injury (Kondratowicz et al., 2011; Yuan et al., 2015; Costafreda et al., 2018), which makes it a more specific and maybe also safer therapeutic target for COVID-19 patients with kidney diseases.

In summary, our data suggest a crucial role of KIM1 in SARS-CoV-2 renal tropism as a potential receptor for SARS-CoV-2. Here, we propose a model of a ‘vicious cycle’ co-mediated by KIM1 and ACE2 (Figure 5G), which may explain the renal tropism of SARS-CoV-2 in COVID-19 patients. During the initial stage of SARS-CoV-2 invasion, the higher physiological level (Supplementary Figure S1) and binding affinity (Supplementary Table S1) make ACE2 the primary target, which is not kidney-specific. However, after onset of virus-induced acute kidney injury, the resulting drastically upregulated KIM1 rapidly promotes a secondary viral infection co-mediated by KIM1 and ACE2, which is more kidney-specific, and consequently exacerbates kidney damage in a vicious cycle (Figure 5G). Approaches that can break the interaction between SARS-CoV-2 and KIM1, including anti-KIM1 antibodies, small-molecule inhibitors, and KIM1-derived antagonist peptides, may shed light on COVID-19 treatment.

## Materials and methods

### *Materials*

Recombinant SARS-CoV-2-RBD (T80302) was obtained from Genscript. Antagonist peptide 1 (AP1, SCSLFTCQNGIV, purity >95%) and antagonist peptide 2 (AP2, SCSLFTCQNGGGWF, purity >95%) were chemically synthesized by Genscript. Anti-mouse-IgG antibody (p/n 18-8816-33) and anti-rabbit-IgG antibody (p/n 18-8817-33) were obtained from RockLand. IgG with SureBeads™ Protein G magnetic beads (J2112LB-02) was purchased from Bio-Rad. DAPI (D9542) was from Sigma. Alex Flour 594 labelled phalloidin (C2205S) was from Beyotimes. Antibodies against KIM1 (NBP1-76701, Novus Biologicals), Flag (F1804, Sigma), HA (H6908, Sigma), and ACE2 (21115-1-AP, Proteintech) were used.

### *Acquisition and analysis of expression profiles of KIM1 and ACE2*

To obtain the comprehensive transcriptome and protein profiles of KIM1 and ACE2 for human tissues, we collected and analyzed the transcriptome data and immunohistochemistry-based protein profiles from Human Protein Atlas (HPA, https://www.proteinatlas.org), which showed the expression and localization of human proteins across tissues and organs, based on deep sequencing of RNA (RNA-seq) from 37 normal tissue and immunohistochemistry on tissue microarrays containing 44 tissue types (Uhlen et al., 2015). HPA RNA-seq tissue of the protein-coding gene was recorded as mean protein-coding transcripts per million (pTPM), corresponding to mean values of samples from each tissue. Histology-based protein expression levels were analyzed manually into 4 levels (not detected, low, medium, and high). In Supplementary Figure S1, top 10 tissular transcriptional levels and histology-based protein expression levels of KIM1 and ACE2 are listed, respectively, and the overlapped expression profile of KIM1 and ACE2 are summarized.

### *Molecular docking and dynamics simulations*

Dockings were conducted via Z-Dock (http://zdock.umassmed.edu/). Crystal structures of SARS-CoV-RBD (PBD ID 2AJF), SARS-CoV-2-RBD (PDB ID 6M0J), MERS-COV-RBD (PDB ID 4L3N), ACE2 (PDB ID 1R42), and KIM1 Ig V domain (PDB ID 5DZO) were used to seek potential binding models. The best-scored protein complexes were selected for the following molecular dynamics simulations, which were conducted by the Desmond server (http://www.deshawresearch.com/) and analyzed by Pymol2.3 and Maestro11.8.012. The 50 nsec dynamics simulations diagram was applied to study the dynamic parameters of the protein complexes. MM-GBSA binding free energy was calculated by HawkDock (Misini Ignjatovic et al., 2016).

*Root mean square deviation (RMSD) and root mean square fluctuation (RMSF)*

RMSD was utilized to estimate the average change in displacement of a selection of atoms for a particular frame as described (Li et al., 2011). RMSF was conducted to study the displacement changes in the protein chain (Li et al., 2011).

*AKI mouse models and qPCR*

I/R injury was performed on C57BL/6 mice as we previously described (Chen et al., 2015, 2017). For cisplatin-induced acute kidney injury, 30 mg/kg bodyweight cisplatin was injected intraperitoneally into 8-week-old male mice, and mice were sacrificed 3 days later. Blood and kidney samples were collected for further analysis, with $n$ = 4 for each experimental animal group. Total RNA was isolated from kidneys by RNA$^{iso}$ Plus (TaKaRa) and reverse-transcribed into cDNA using the M-MLV first-strand synthesis system (Invitrogen). The abundance of specific gene transcripts was assessed by qPCR. Primers used in the study are provided (Supplementary Table S4).

*Constructs*

Mammalian expression plasmids for human KIM1, KIM1 Ig V (KIM1 residues aa 20–127), KIM1 ΔIg V (truncated KIM1 without residues aa 20–127), KIM1-CFP, KIM1 Ig V-CFP, ACE2, SARS-CoV-2-RBD (SARS-CoV-2-S residues aa 319–541), SARS-CoV-2-S, SARS-CoV-2-RBD-YFP, and TIM4-YFP were constructed. PCR amplification products of the corresponding cDNA fragments were cloned into a pRK promoter-based vector containing either HA or FLAG tag. SARS-CoV-2-related plasmids were kind gifts from Dr P.H. Wang at Shandong University.

*Cell culture and transfection*

Human kidney tubular cell line HK-2 (obtained from China Center for Type Culture Collection) was cultured in DMEM/F12 media (Hyclone) containing 17.5 mM glucose and 10% fetal bovine serum. To evaluate the impact of SARS-CoV-2 on cells, HK-2 cells were transfected with SARS-CoV-2-S

and SARS-CoV-2-RBD plasmids, and then collected for further detection.

*Co-IP*

Indicated HK-2/HEK293T cells ($1\times10^7$) were lysed in 1 ml pre-lysis buffer (25 mM Tris–HCl, pH 7.4, 150 mM NaCl, 1% NP-40, 1 mM EDTA, 5% glycerol), which is formulated for pulldown and IP assays and as a wash buffer for beads. For IP, cell lysate was immunoprecipitated with the indicated antibody or respective IgG with SureBeads™ Protein G magnetic beads overnight at 4°C. After washing with pre-lysis buffer containing 500 mM NaCl, the beads were boiled in loading buffer and subjected to immunoblotting (Wan et al., 2017).

*FRET assay*

Intracellular interaction between KIM1 and SARS-CoV-2-RBD was detected by a standard FRET-based assay (Karpova et al., 2006) using KIM1-CFP and SARS-CoV-2-RBD-YFP. Briefly, mammalian expression plasmids expressing KIM1-CFP or KIM1 ΔIg V-CFP were co-transfected with SARS-CoV-2-RBD-YFP into HEK-293T cells. For fluorescence spectrophotometer-based detection, cells were collected and lysed 24 h after transfection, and the lysate was detected by a F-2700 Fluorescence Spectrophotometer (Hitachi) via wavelength scan (500–600 nm) and time scan (435/527 nm, excitation/emission). For confocal-based detection, cells were imaged with a Leica TCS SP8 confocal microscope under high power objective lens (40×) (CFP channel: 435/485 nm, excitation/emission; YFP channel: 485/527 nm, excitation/emission; FRET channel: 435/527 nm, excitation/emission) (Li et al., 2020b). Co-transfection of unconjugated CFP and YFP was included as a negative control as described (Karpova and McNally, 2006). Interaction between KIM1 and its ligand TIM4 was detected by FRET assay as a positive control (Rong et al., 2011).

*CRISPR–Cas9-mediated knockout of KIM1*

The CRISPR–Cas9-based protocols for genome engineering were used as described (Zhang et al., 2017). Guide RNA target sequences for KIM1 are provided (Supplementary Table S4).

*FITC labelling and confocal microscopy*

FITC label was performed as we previously described (Zhang et al., 2020; Li et al., 2020b). Briefly, SARS-CoV-2-RBD was co-incubated with FITC (molar ratio 1:5) overnight, and then 5 mM $NH_4Cl$ was added to stop the reaction and quench the un-reacted FITC. The solution was dialyzed twice and lyophilized for further use.

HEK293T cells (5 × $10^6$) or HK-2 cells (1 × $10^7$) were incubated with free FITC or FITC-SARS-CoV-2-RBD (100 μg/ml) for 2 . For peptide-based internalization assays, AP1 or AP2 (50 μM) was co-added with FITC-SARS-CoV-2-RBD (100 μg/ml). After fixing with 4% (*w*/*v*) formaldehyde, cell membranes were stained with Alex Flour 594 labelled phalloidin (2 μg/ml) and the nuclei were stained by DAPI (1 μg/ml), and then imaged with a Leica TCS SP8 confocal microscope. For each group, at least 100 cells from 5 fields under high power objective lens (64×) were included in the assessment. Representative images were presented. Quantification of images was conducted by Image J 1.8.0.

*Cell viability assays*

Cells were plated at 3000–4000 cells per well in 96-well plates. At 80% confluence, cells incubated with SARS-CoV-2-RBD (100 μg/ml) were treated with or without AP1 or AP2 (10, 50, 100 μM). After that, 10 μl MTT (5 mg/ml) was added to each well for 4 h, medium was removed, and DMSO was added. Absorbance measured at 490 nm was normalized to the respective control group.

*Statistical analysis*

Data were expressed as mean ± SD. Significant differences were assessed by two-tailed Student's test. Two-sided *P*-value <0.05 was considered statistically significant. Analyses were performed with EXCEL 2017 and GraphPad Prism 8.0.

**Acknowledgements**

The work was technically supported by the Analytical and Testing Center of Huazhong University of Science and Technology.

**Funding**

This work was supported by the Natural Science Foundation of China (31671195 and 31971066), the Integrated Innovative Team for Major Human Diseases Program of Tongji Medical College, the Front Youth Program of HUST, and Innovation Funding Project of HUST (2020yjsCXCY042).

**Author contributions:** K.H. designed the study. C.Y., Y.Z., X.Z., H.-J.C., Y.C., D.Y., Z.S., X.W., X.L., M.X., and H.C. performed the experiments. C.Y., Y.Z., H.C., and K.H. prepared the manuscript.

**Conflict of interest:** none declared.

**Table 1 MM-GBSA binding free energy of residues in SARS-CoV-2-RBD and KIM1 Ig V complex.**

| Rank | SARS-CoV-2-RBD | | KIM1 Ig V | |
|---|---|---|---|---|
| | Residue | Binding free energy (kcal/mol) | Residue | Binding free energy (kcal/mol) |
| 1 | Val367 | -2.42 | Leu54 | -6.14 |
| 2 | Trp436 | -2.00 | Trp112 | -5.26 |
| 3 | Phe374 | -1.86 | Leu54 | -4.96 |
| 4 | Ser371 | -1.84 | Phe113 | -3.97 |
| 5 | Phe338 | -1.53 | Gln58 | -2.11 |
| 6 | Leu368 | -1.50 | Asn114 | -0.75 |
| 7 | Asn437 | -1.46 | Asn59 | -0.13 |
| 8 | Asn440 | -1.46 | Asp115 | -0.13 |
| 9 | Ser373 | -1.18 | Asp74 | -0.12 |
| 10 | Leu335 | -0.96 | Asp99 | -0.11 |

Top 10 ranked residues involved in the binding of SARS-CoV-2-RBD and KIM1 Ig V are listed.

**Figure legends**

**Figure 1** Basic information of SARS-CoV-2-S and KIM1. (**A**) Structural scheme of SARS-CoV-2-S. NTD, N-terminal domain; RBM, receptor-binding motif; SD1, subdomain 1; SD2, subdomain 2; FP, fusion peptide; HR1, heptad repeat 1; HR2, heptad repeat 2; TM, transmembrane region; IC, intracellular domain. The domain boundaries of TM and IC have not been precisely defined, and thus the residues are not labelled. (**B**) Sequence alignment of SARS-CoV-RBD, SARS-CoV-2-RBD, and MERS-CoV-RBD. ACE2-contacting residues of SARS-CoV-RBD and SARS-CoV-2-RBD are highlight in yellow; KIM1-contacting residues of SARS-CoV-RBD are in green; KIM1-contcacting residues of SARS-CoV-RBD-2 are in blue; asterisks indicate fully conserved residues; colons indicate partly-conserved residues; periods indicate weakly conserved residues. (**C**) Relative mRNA levels of ACE2 and KIM1 from the kidneys of I/R- and cisplatin-induced kidney injury mouse models. $^{*}P < 0.05$, $^{**}P < 0.01$; ns, no significance. (**D** and **E**) Structural scheme of KIM1 domains, in relation to cell membrane. Signal, signal peptide region; Mucin, mucin-containing domain.

**Figure 2** Binding model of SARS-CoV-2-RBD and KIM1 Ig V. (**A**) Low-energy binding conformations of SARS-CoV-2-RBD binding to KIM1 Ig V. Left panel: the surface model of SARS-CoV-2-RBD. Right panel: high-resolution image of the binding sites, Phe338, Val367, Ser371, Phe374, and Trp436 of SARS-CoV-2-RBD interacting with Leu54, Phe55, Gln58, Trp112, and Phe113 of KIM1 Ig V. (**B**) Distinct binding regions of KIM1 and ACE2 in SARS-CoV-2-RBD, with KIM1-binding pocket in red and ACE-2 binding pocket in blue. (**C**) SARS-CoV-RBD and SARS-CoV-2-RBD bind with the same pocket of KIM1 Ig V.

**Figure 3** SARS-CoV-2-RBD binds with KIM1 Ig V. (**A**) Constructs used in co-IP studies. (**B**) The interaction between overexpressed Flag-tagged spike/RBD and HA-tagged KIM1 in HEK293T cells. The indicated plasmids were co-transfected into HEK293T ($1\times10^7$). After 24 h, cells were lysed and subjected to co-IP followed by immunoblotting with indicated antibodies. (**C**) The interaction between KIM1 Ig V domain and SARS-CoV-2-RBD in KIM1 knockout HK-2 cells. For IP group, KIM1 and KIM1 Ig V domain were detected by anti-KIM1 antibody. Mammalian expression plasmids encoding Flag-tagged spike/RBD were transfected to KIM1 knockout HK-2 cells ($1\times10^7$). After 36 h, cells were lysed and subjected to co-IP followed by immunoblotting with indicated antibodies. Anti-rabbit light chain-specific IgG was used to avoid interference of IgG heavy chain. (**D**) The interaction between KIM1 Ig V domain and SARS-CoV-2-RBD in HEK293T cells. The experiments were performed as in **B** except that mammalian expression plasmids encoding

HA-tagged KIM1, KIM1 Ig V domain, and truncated KIM1 without Ig V domain (ΔIg V) were used. Anti-rabbit light chain-specific IgG was used to avoid interference of IgG heavy chain. (**E**) FRET signals of KIM1 and SARS-CoV-2-RBD detected by confocal microscopy. Unconjugated CFP and YFP were co-transfected as the negative control, and the interaction between KIM1 and its ligand TIM4 was included as a positive control. CFP channel: 435/485 nm, excitation/emission; YFP channel: 485/527 nm, excitation/emission; FRET channel: 435/527 nm, excitation/emission. Scale bar, 1.5 μm. (**F–I**) Detection of FRET signals using fluorescent wavelength scan for unconjugated CFP and YFP (**F**), KIM1-CFP and TIM4-YFP (**G**), KIM1-CFP and SARS-CoV-2-RBD-YFP (**H**), and KIM1 ΔIg V-CFP and SARS-CoV-2-RBD-YFP (**I**). (**J**) Quantitative FRET intensity of the indicated four groups. $^{*}P < 0.05$, $^{**}P < 0.01$; ns, no significance.

**Figure 4** KIM1 mediates the cell entry of SARS-CoV-2-RBD. (**A**) Representative images and quantitative data of cell surface attachment of SARS-CoV-2-RBD in wild-type (WT) and KIM1 knockout (KIM1-KO) HK-2 cells. Scale bar, 20 μm. $^{***}P < 0.001$. (**B**) Representative images and quantitative data of cell surface attachment of SARS-CoV-2-RBD in HEK293T cells. Scale bar, 20 μm. OE KIM1, overexpression of KIM1. $^{**}P < 0.01$.

**Figure 5** Rationally designed AP2 inhibits the cell entry of SARS-CoV-2. (**A**) Schematic diagram of AP1 and AP2. (**B**) Effects of AP1 and AP2 on the cell attachment of SARS-CoV-2-RBD Scale bar, 20 μm. (**C**) Quantitative analysis of the cell attachment of SARS-CoV-2-RBD upon administration of AP1 and AP2. $^{*}P < 0.05$; ns, no significance. (**D**) Protective effects of AP2 against SARS-CoV-2-RBD. $^{**}P < 0.01$; $^{\#\#\#}P < 0.001$ compared to RBD + 10 μM AP2 group; ns, no significance. (**E**) FRET signal between KIM1 and SARS-CoV-2-RBD in the presence or absence of AP1 and AP2. (**F**) Quantitative FRET intensity of KIM1-CFP and SARS-CoV-2-RBD-YFP in the presence of AP1 and AP2. $^{*}P < 0.05$, $^{**}P < 0.01$; ns, no significance. (**G**) A proposed working model of 'a vicious cycle' co-mediated by KIM1 and ACE2 in the kidney of COVID-19 patients. KIM1/ACE2 mediate the initial kidney infection, and the resulting acute kidney injury drastically upregulates KIM1, which in turn promotes infection and consequently exacerbates the kidney injury. KIM1-derived antagonist peptide may competitively bind with SARS-CoV-2-RBD to intervene viral invasion.

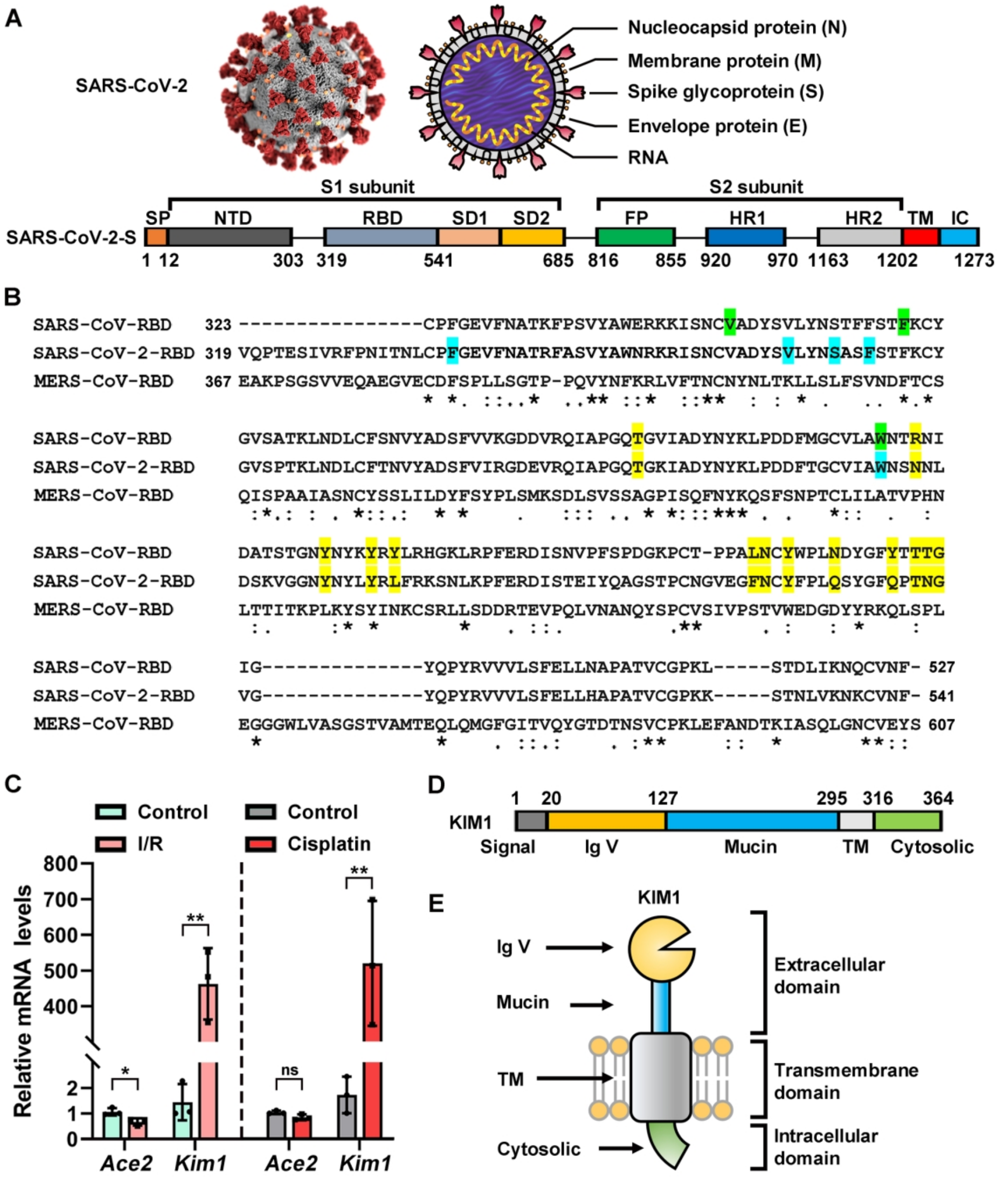

Figure 1

170x199mm (300 x 300 DPI)

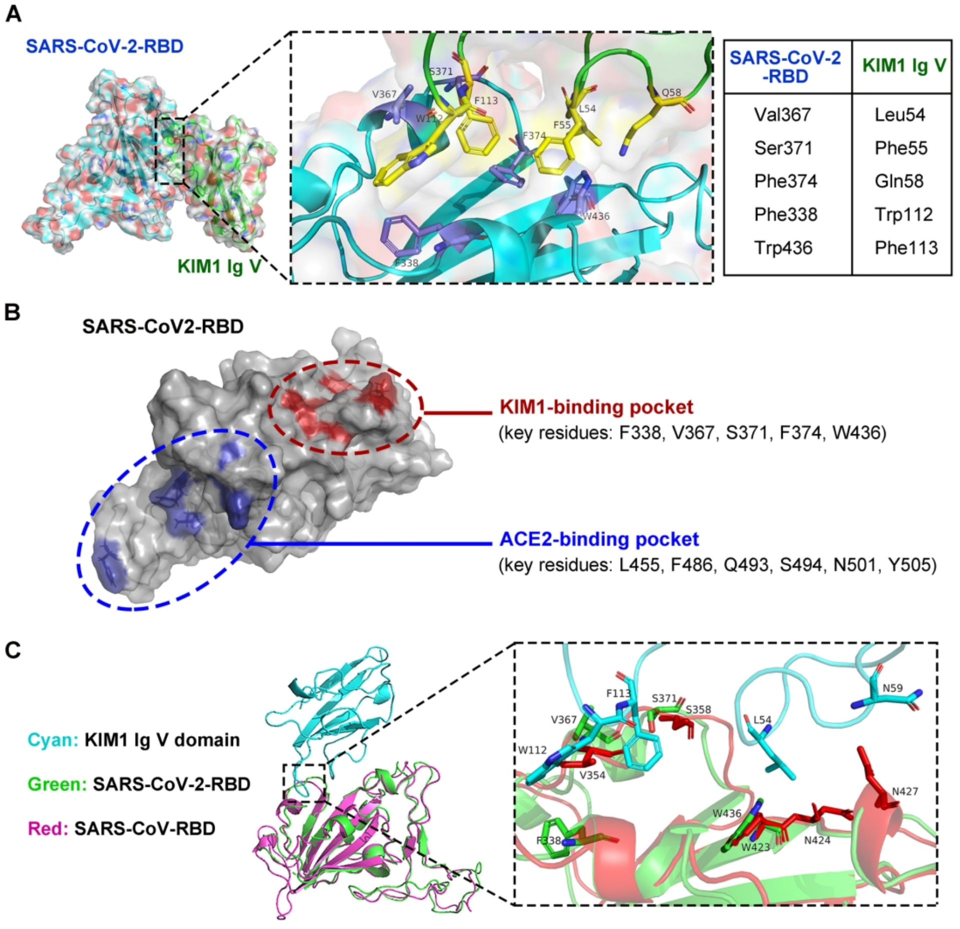

Figure 2

180x199mm (300 x 300 DPI)

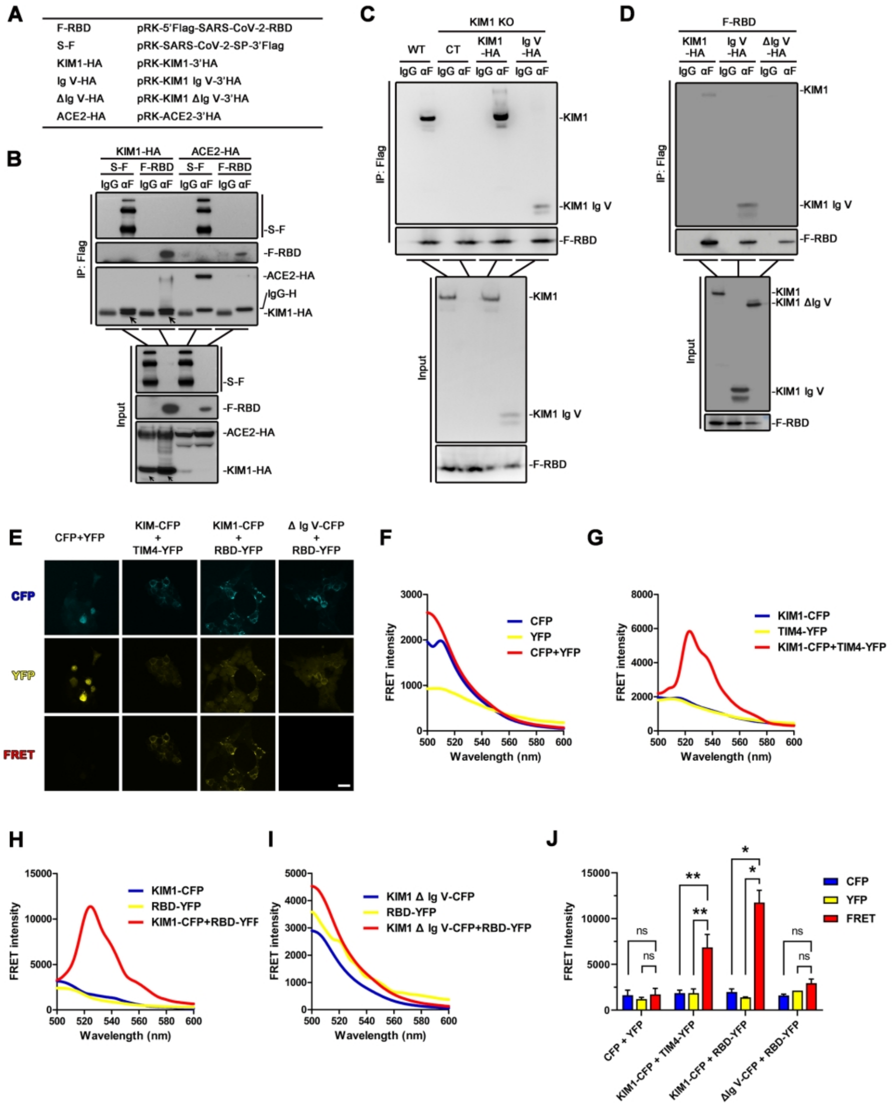

Figure 3

180x240mm (300 x 300 DPI)

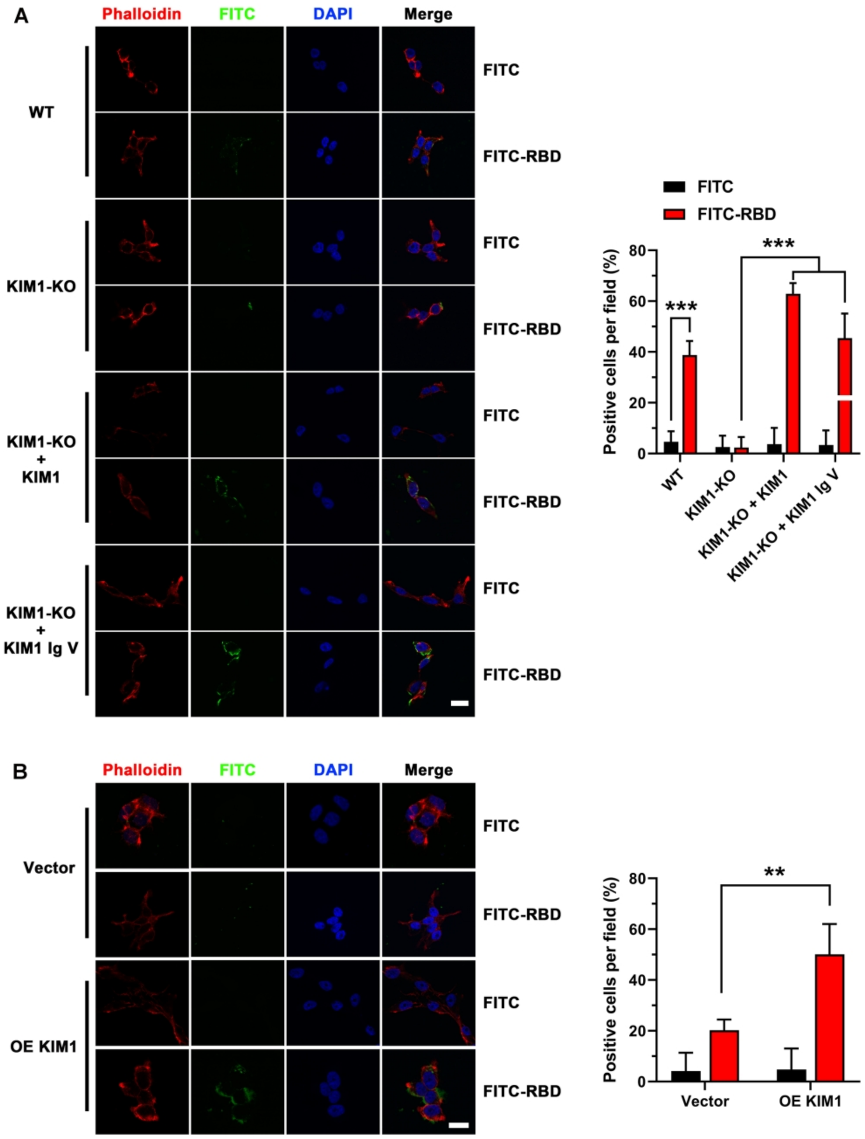

Figure 4

180x240mm (300 x 300 DPI)

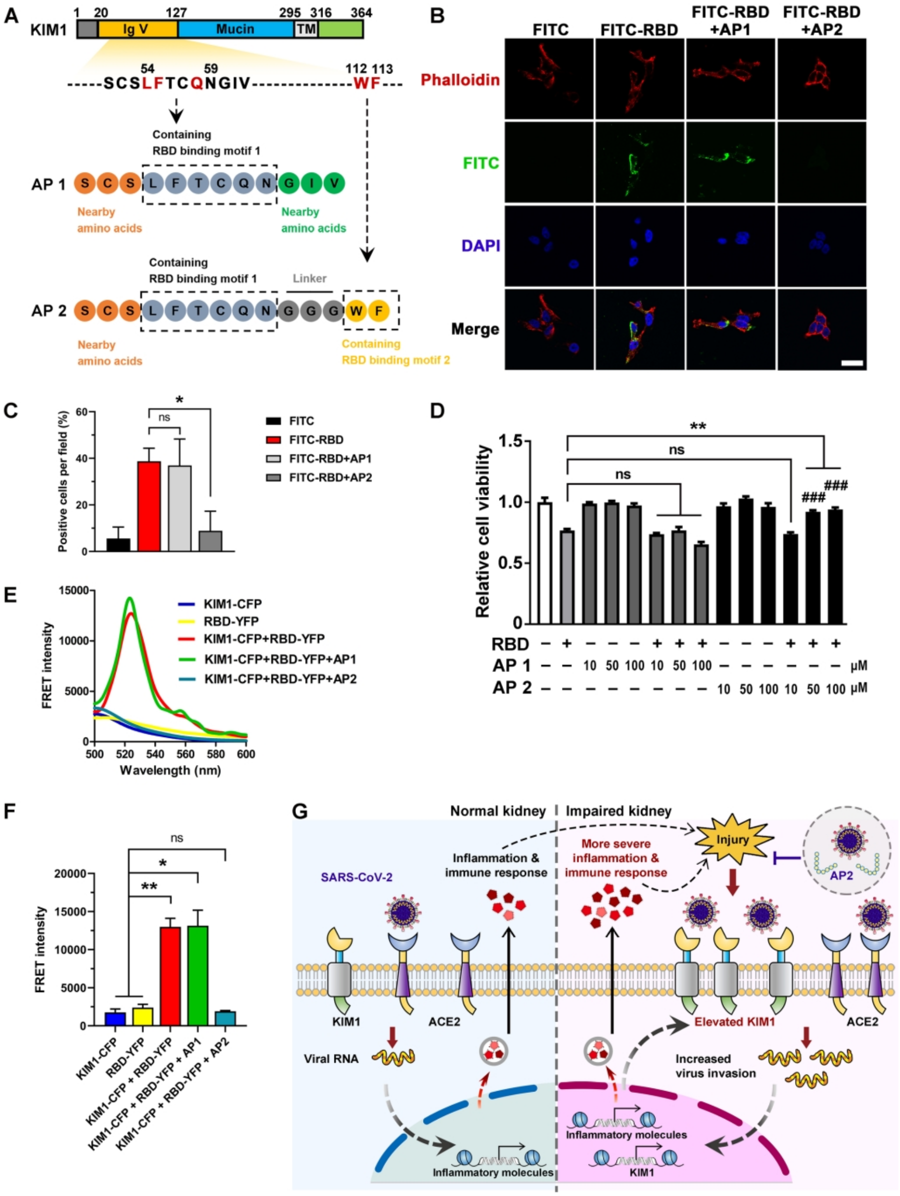

Figure 5

180x240mm (300 x 300 DPI)
