# Supplementary material for: Kidney injury molecule-1 is a potential receptor for SARS-CoV-2
Source: J Mol Cell Biol. 2021 Jan 25;13(3):185–96. doi: 10.1093/jmcb/mjab003 (PMC7928767; doi:10.1093/jmcb/mjab003)
Supplement: mjab003_Supplementary_Data [file mjab003_supplementary_data.pdf]

Supplementary material

for

Kidney injury molecule-1 is a potential receptor for SARS-CoV-2

Short title: KIM1 mediates SARS-CoV-2 invasion

Chen Yang[1,†], Yu Zhang[1,†], Xia Zeng[1], Huijing Chen[1], Yuchen Chen[1], Dong Yang[1], Ziwei Shen[1], Xiaomu Wang[1], Xinran Liu[1], Mingrui Xiong[1], Hong Chen[1,*], and Kun Huang[1,2,*]

[1] School of Pharmacy, Tongji Medical College, Huazhong University of Science & Technology, Wuhan 430030, China

[2] Tongji-RongCheng Biomedical Center, Tongji Medical College, Huazhong University of Science & Technology, Wuhan 430030, China

[†] These authors contributed equally to this work.

* Correspondence to: Hong Chen, E-mail: hongchen2017@hust.edu.cn; Kun Huang, E-mail: kunhuang@hust.edu.cn

Table of contents

Supplementary Table S1 MM-GBSA binding free energy of SARS-CoV-2-RBD and receptors.

Supplementary Table S2 Top 20 clinically identified mutations in SARS-CoV-2 spike protein.

Supplementary Table S3 MM-GBSA binding free energy of residues in SARS-CoV-RBD and KIM1 Ig V complex.

Supplementary Table S4 Primers used in the study.

Supplementary Figure S1 Expression profiles of KIM1, ACE2 and molecular dynamics docking information of SARS-CoV-2-RBD and KIM1.

Supplementary Figure S2 Molecular dynamics simulations information of SARS-CoV-2-RBD and KIM1.

Supplementary Figure S3 Clinically identified mutations in SARS-CoV-2 spike protein

Supplementary Figure S4 Molecular dynamics simulations information of SARS-CoV-RBD and KIM1.

Supplementary Figure S5 Binding model of SARS-CoV-RBD and KIM1.

Supplementary Figure S6 Identification of KIM1 knockout HK-2 cell line and the protective effects of antagonist peptide on SARS-CoV-2-RBD induced cytotoxicity.

Supplementary Table S1 MM-GBSA binding free energy of SARS-CoV-2-RBD and receptors.

Ligand	Receptor	Binding free energy (kcal/mol)
SARS-CoV-RBD	KIM1 Ig V domain	-21.59
SARS-CoV-2-RBD	KIM1 Ig V domain	-35.64
SARS-CoV-2-RBD (V367F)	KIM1 Ig V domain	-37.26
SARS-CoV-2-RBD	ACE2	-50.60
MERS-COV-RBD	KIM1 Ig V domain	-10.12
SARS-CoV-2-RBD	AP1	-7.13
SARS-CoV-2-RBD	AP2	-6.65

MM-GBSA binding free energy of the protein complex was cauclated by the accumulation of the binding free energy of all involved residues. The binding free energy of SARS-CoV-2-RBD and ACE2 was also cauclated and provided as a reference.

Supplementary Table S2 Top 20 clinically identified mutations in SARS-CoV-2 spike protein.

Rank	Mutation	Occurrence (Log10)	Rank	Mutation	Occurrence (Log10)
1	D614G	5.016	11	<u>D294D</u>	3.004
2	<u>Q616Q</u>	3.906	12	<u>P25P</u>	2.961
3	S477N	3.886	13	D936Y	2.940
4	<u>T723T</u>	3.710	14	<u>N824N</u>	2.929
5	A222V	3.322	15	<u>F275F</u>	2.892
6	<u>Y789Y</u>	3.163	16	P1263L	2.866
7	<u>F306F</u>	3.155	17	<u>P715P</u>	2.779
8	R21I	3.050	18	L54F	2.676
9	L5F	3.037	19	D253G	2.646
10	N439K	3.004	20	<u>T1100T</u>	2.598

Synonymous mutations are underlined. Data were acquired on October 5, 2020 from http://giorgilab.dyndns.org/coronapp/.

Supplementary Table S3 MM-GBSA binding free energy of residues in SARS-CoV-RBD and KIM1 Ig V domain complex.

Rank	SARS-CoV-RBD		KIM1 Ig V	
	Residue	Binding free energy (kcal/mol)	Residue	Binding free energy (kcal/mol)
1	Phe360	-3.67	Trp112	-4.87
2	Val354	-1.93	Phe55	-4.33
3	Asn424	-1.34	Leu54	-4.17
4	Trp423	-1.12	Phe113	-3.62
5	Asn427	-0.71	Gln58	-1.46
6	Thr359	-0.69	Asn114	-1.42
7	Ser358	-0.68	Asn59	-0.54
8	Phe361	-0.59	Asp115	-0.37
9	Leu355	-0.45	Asp74	-0.20
10	Asn357	-0.41	Asp99	-0.20

Top 10 ranked residues that involved in the binding of SARS-CoV-RBD and KIM1 Ig V domain were listed.

Supplementary Table S4 Primers used in the study.

Primer	Sequence: 5' to 3'
**Primers for qPCR**	
M-*Ace2*-F	CAAGTGTTGGCTTCGGTGTG
M-*Ace2*-R	ATTCAAGTGACCAGCGAGCA
M-*Kim1*-F	TCCACACATGTACCAACATCAA
M-*Kim1*-R	GTCACAGTGCCATTCCAGTC
M-*Actinb*-F	GGCTGTATTCCCCTCCATCG
M-*Actinb*-R	CCAGTTGGTAACAATGCCATGT
**Primers for CRISPR-Cas9 system**	
H-*KIM1*-KO-1-F	CACCGCTGACGGCCAATACCACTAA
H-*KIM1*-KO-1-R	AAACTTAGTGGTATTGGCCGTCAGC
H-*KIM1*-KO-2-F	CACCGGTTCGAACAGTCGTGACGGT
H-*KIM1*-KO-2-R	AAACACCGTCACGACTGTTCGAACC
H-*KIM1*-KO-3-F	CACCGGTCGTTGGAACAGTCGTCAT
H-*KIM1*-KO-3-R	AAACATGACGACTGTTCCAACGACC

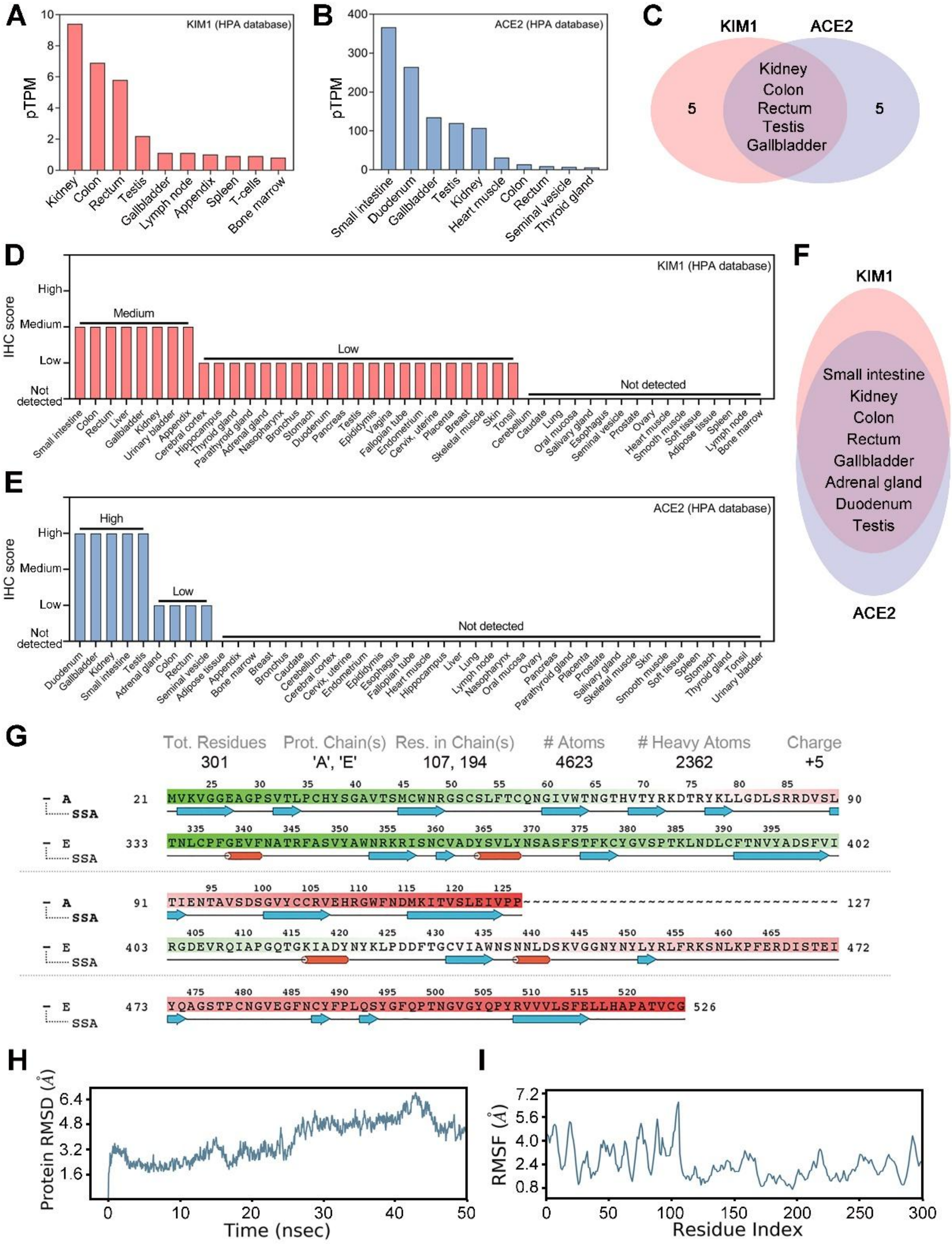

Supplementary Figure S1 Expression profiles of KIM1, ACE2 and molecular dynamics docking information of SARS-CoV-2-RBD and KIM1. (**A**) Tissue transcriptional expression of *KIM1* from The Human Protein ATLAS dataset (HPA). pTPM, protein-coding transcripts per million. (**B**) Tissue transcriptional expression of *ACE2* from HPA. (**C**) Transcriptional expression intersection of *KIM1* and *ACE2* form HPA. (**D**) Histology-based protein expression levels of KIM1. (**E**) Histology-based protein expression levels of ACE2. (**F**) Histology-based protein expression intersection of KIM1 and

ACE2. (**G**) Basic protein information of SARS-CoV-2-RBD and KIM1 Ig V domain. Red cylinder represents α-Helix structure. Blue arrow indicates β-Sheet structure. (**H**) RMSD values of the SARS-CoV-2-RBD and KIM1 Ig V domain binding models. (**I**) RMSF values of the SARS-CoV-2-RBD and KIM1 Ig V domain binding models.

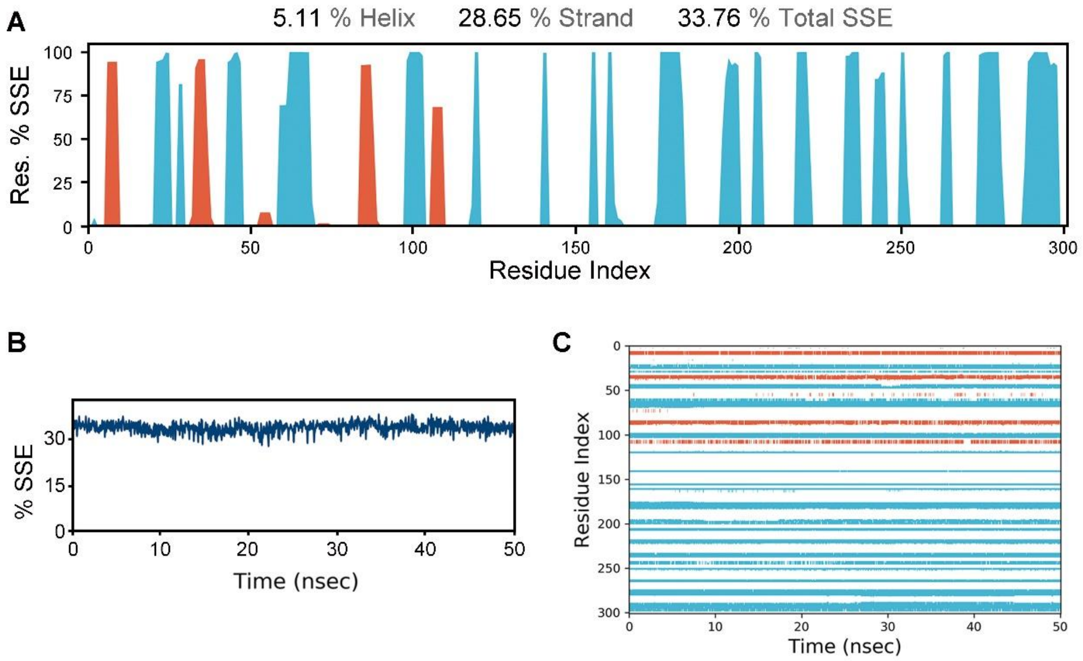

Supplementary Figure S2 Molecular dynamics simulations information of SARS-CoV-2-RBD and KIM1. (**A**) Residue SSE distribution by residue index throughout the protein structure. Red column suggests α-Helix structure and blue colunm indicates β-strand structure. (**B**) SSE composition for trajectory frames in the dynamics simulation process of SARS-CoV-2-RBD and KIM1 Ig V domain, the curve indicates SSE composition for each trajectory frame over the course of the simulation. (**C**) SSE assignment of the residues over the simulation time, red dots indicates α-Helix structures and blue dots indicate β-strand structures. SSE, sencondary structure elements.

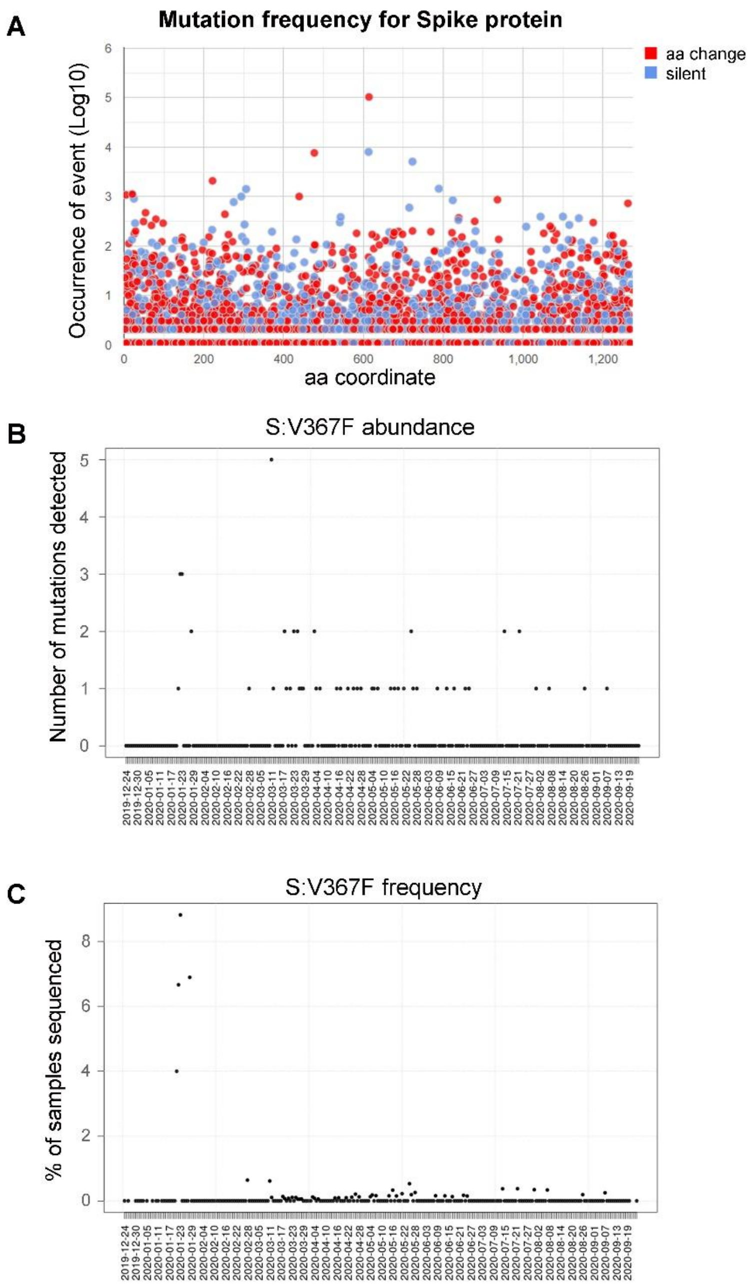

Supplementary Figure S3 Clinically identified mutations in SARS-CoV-2 spike protein. (**A**) Mutation frequency for SARS-CoV-2 spike protein worldwide. Blue dots indicate synonymous mutation and red dots refer to amino acid substitution. (**B**) The number of clinically detected V367F mutation in COVID-19 patients. (**C**) Percentage of COVID-19 cases carrying V367F mutation. Data were acquired on October 5, 2020 from http://giorgilab.dyndns.org/coronapp/.

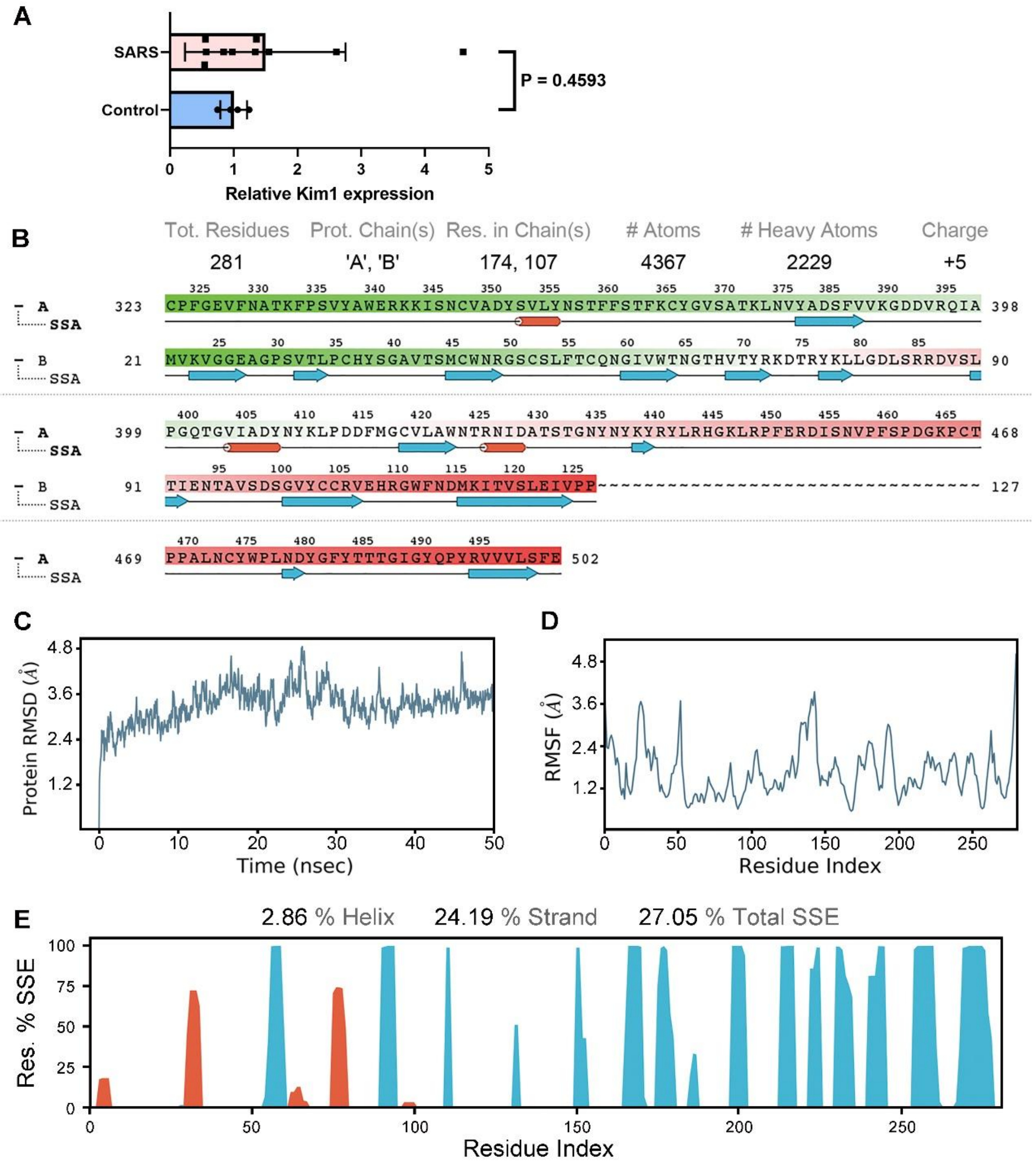

Supplementary Figure S4 Molecular dynamics simulations information of SARS-CoV-RBD and KIM1. (**A**) *KIM1* mRNA expression in 10 SARS patients-derived peripheral blood mononuclear cells and 4 control samples (GSE1739). (**B**) Basic protein information of SARS-CoV-RBD and KIM1 Ig V domain. Red cylinder represents α-Helix structure of, blue arrow indicates β-Sheet structure. (**C**) RMSD values of the SARS-CoV-RBD and KIM1 Ig V domain binding models. (**D**) RMSF values of SARS-CoV-RBD and KIM1 Ig V domain. (**E**) Residue SSE distribution by residue index throughout the protein structure. Red column suggests α-Helix structure and blue colunm indicates β-strand structure. SSE, secondary structure elements.

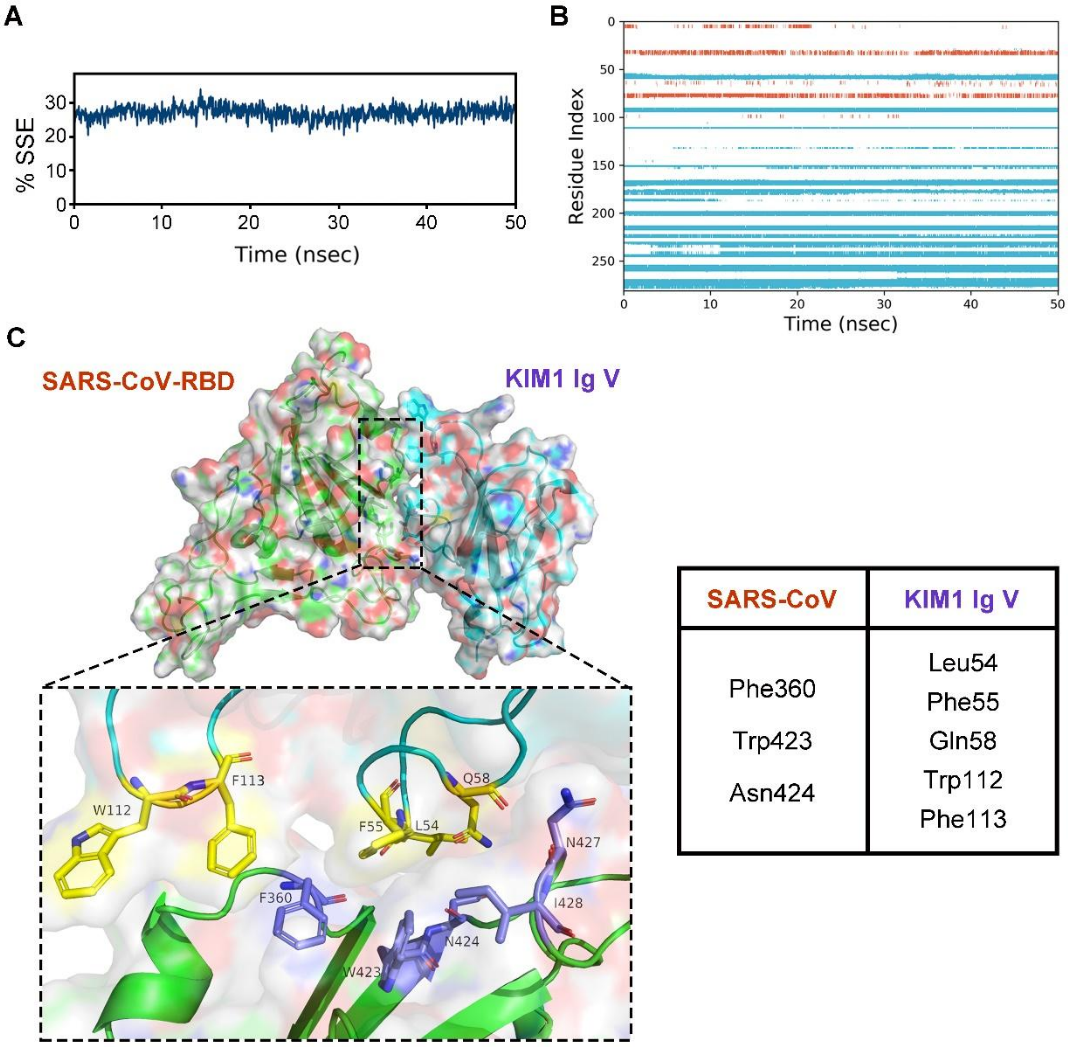

SARS-CoV	KIM1 Ig V
Phe360<br>Trp423<br>Asn424	Leu54<br>Phe55<br>Gln58<br>Trp112<br>Phe113

Supplementary Figure S5 Binding model of SARS-CoV-RBD and KIM1. (**A**) SSE composition for trajectory frames in the dynamics simulation process of SARS-CoV-RBD and KIM1 Ig V domain, the curve indicates SSE composition for each trajectory frame over the course of the simulation. (**B**) SSE assignment of the residues over the simulation time, red dots indicate α-Helix structures and blue dots indicate β-strand structures. (**C**) Low-energy binding conformations of SARS-CoV-RBD binds to KIM1 Ig V domain. SSE, secondary structure elements.

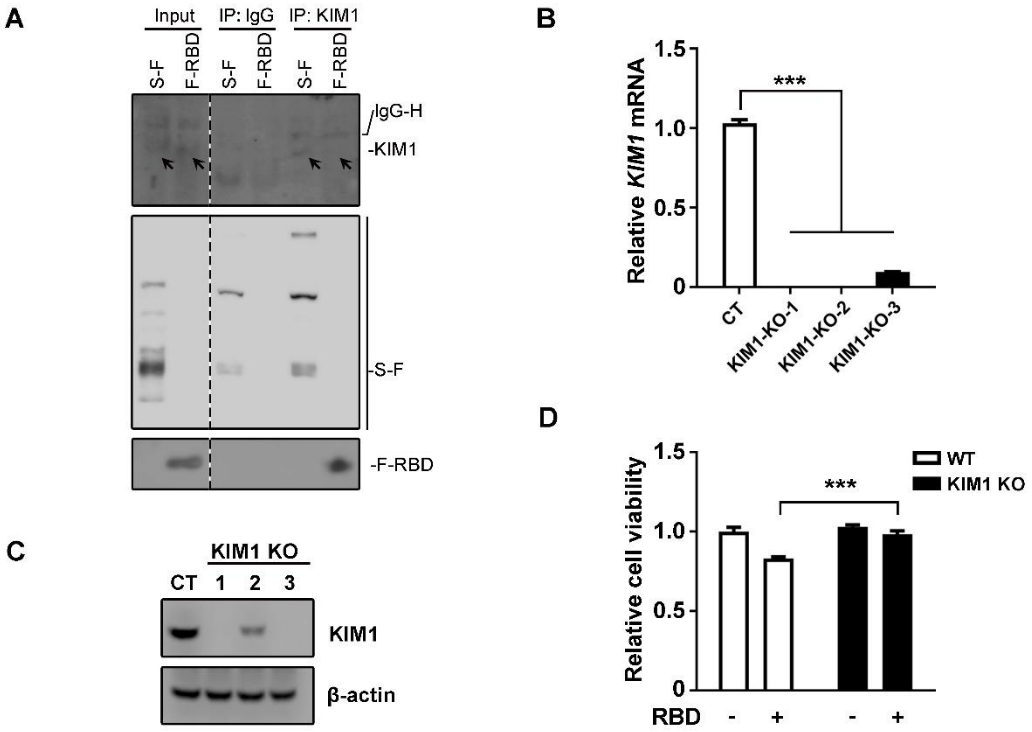

Supplementary Figure S6 Identification of KIM1 knockout HK-2 cell line and the protective effects of antagonist peptide on SARS-CoV-2-RBD induced cytotoxicity. (**A**) The interaction between endogenous KIM1 and overexpressed Flag-tagged Spike/RBD proteins in HK-2 cells. Mammalian expression plasmids encoding Flag-tagged Spike/RBD were transfected to HK-2 cells (1×10^7). 36 hours later, cells were lysed and subjected to co-immunoprecipitation and followed by immunoblotting with indicated antibodies. IgG-H indicates the heavy chain of IgG. (**B**) *KIM1* mRNA expression in WT and KIM1 knockout HK-2 cell line. (**C**) KIM1 protein level in WT and KIM1 knockout HK-2 cell line. (**D**) Cytotoxicity of SARS-CoV-2-RBD in WT and KIM1 knockout HK-2 cell line. $^{***}P < 0.001$.
